# Retraction: Spatial variations in the biochemical potential of okra [*Abelmoschus esculentus* L. (Moench)] leaf and fruit under field conditions

**DOI:** 10.1371/journal.pone.0274199

**Published:** 2022-09-14

**Authors:** The PLOS ONE Editors

The *PLOS ONE* Editors retract this article [1] because it was identified as one of a series of submissions for which we have concerns about authorship, competing interests, and peer review. We regret that the issues were not addressed prior to the article’s publication.

NAA and SA did not agree with the retraction. SS, MHS, SZ, SMA, MHA, and AA either did not respond directly or could not be reached.

## References

[1] Sarwar S, Akram NA, Saleem MH, Zafar S, Alghanem SM, Abualreesh MH, et al. (2022) Spatial variations in the biochemical potential of okra [*Abelmoschus esculentus* L. (Moench)] leaf and fruit under field conditions. PLoS ONE 17(2): e0259520. 10.1371/journal.pone.0259520 35113880 PMC8812902

